# Supplementary material for: Identifying Candidate Gene Drivers Associated with Relapse in Pediatric T-Cell Acute Lymphoblastic Leukemia Using a Gene Co-Expression Network Approach
Source: Cancers (Basel). 2024 Apr 25;16(9):1667. doi: 10.3390/cancers16091667 (PMC11083586; doi:10.3390/cancers16091667)
Supplement: Supplementary file 1 [file cancers-16-01667-s001.zip › Supplemental Figures_WGCNA.pptx]

## Slide 1
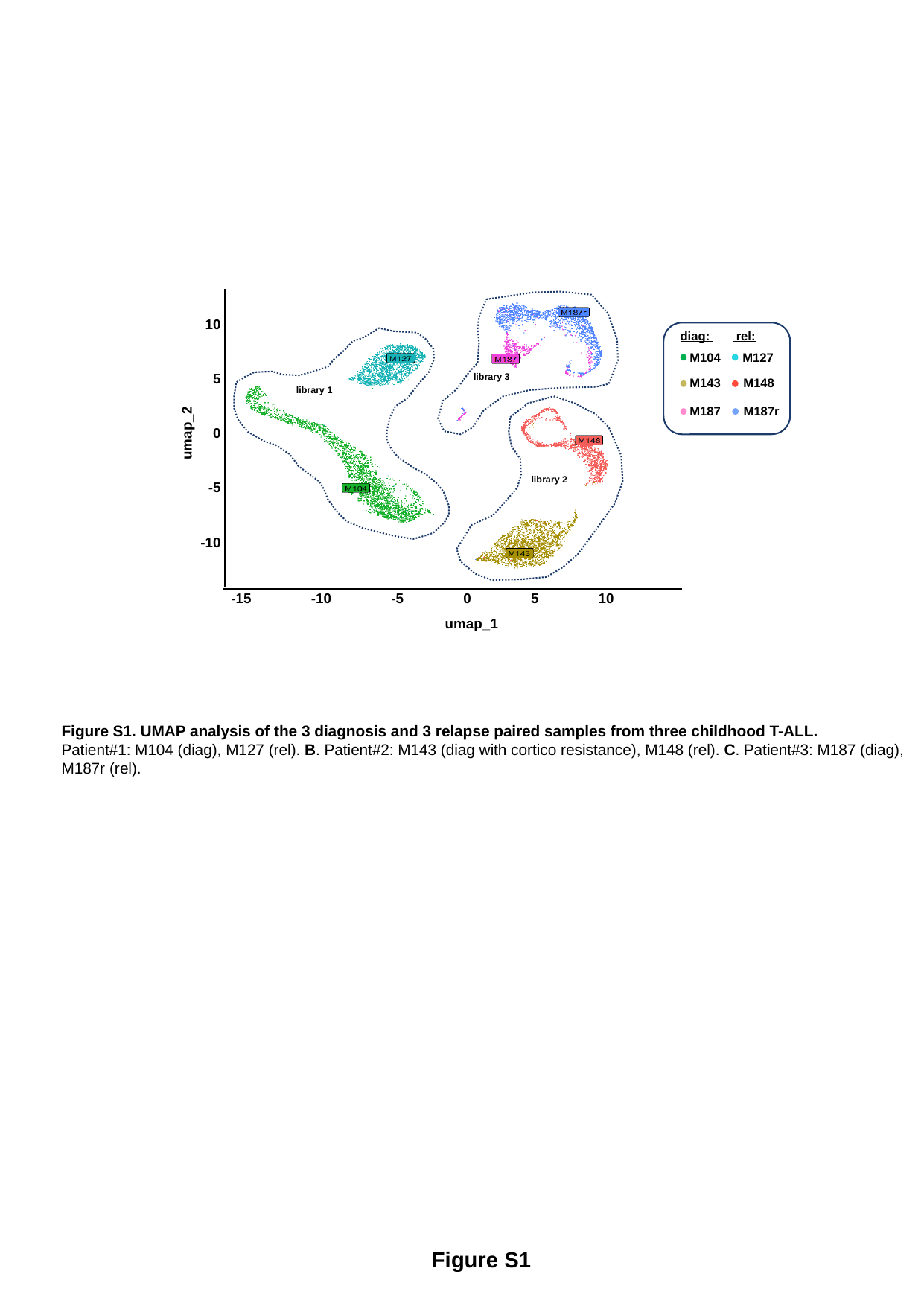

10
diag:
 rel:
M104
M127
5
M143
M148
M187
M187r
umap_2
0
-5
-10
-15
-10
-5
0
5
10
umap_1
library 3
library 1
library 2
Figure S1. UMAP analysis of the 3 diagnosis and 3 relapse paired samples from three childhood T-ALL.
Patient#1: M104 (diag), M127 (rel). B. Patient#2: M143 (diag with cortico resistance), M148 (rel). C. Patient#3: M187 (diag), M187r (rel).
Figure S1

## Slide 2
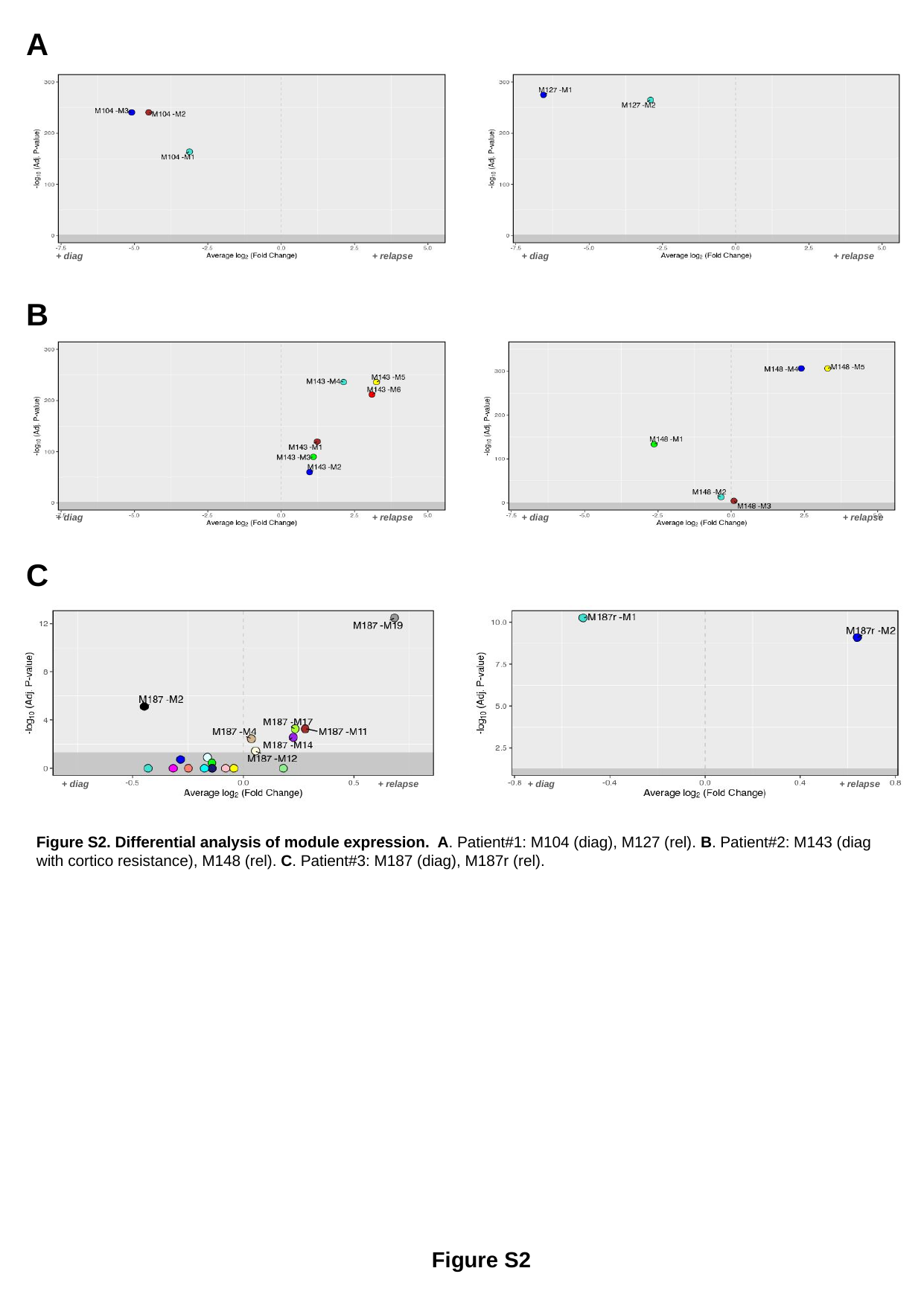

A
+ diag
+ relapse
+ diag
+ relapse
B
+ diag
+ relapse
+ diag
+ relapse
C
+ diag
+ relapse
+ diag
+ relapse
Figure S2. Differential analysis of module expression. A. Patient#1: M104 (diag), M127 (rel). B. Patient#2: M143 (diag with cortico resistance), M148 (rel). C. Patient#3: M187 (diag), M187r (rel).
Figure S2

## Slide 3
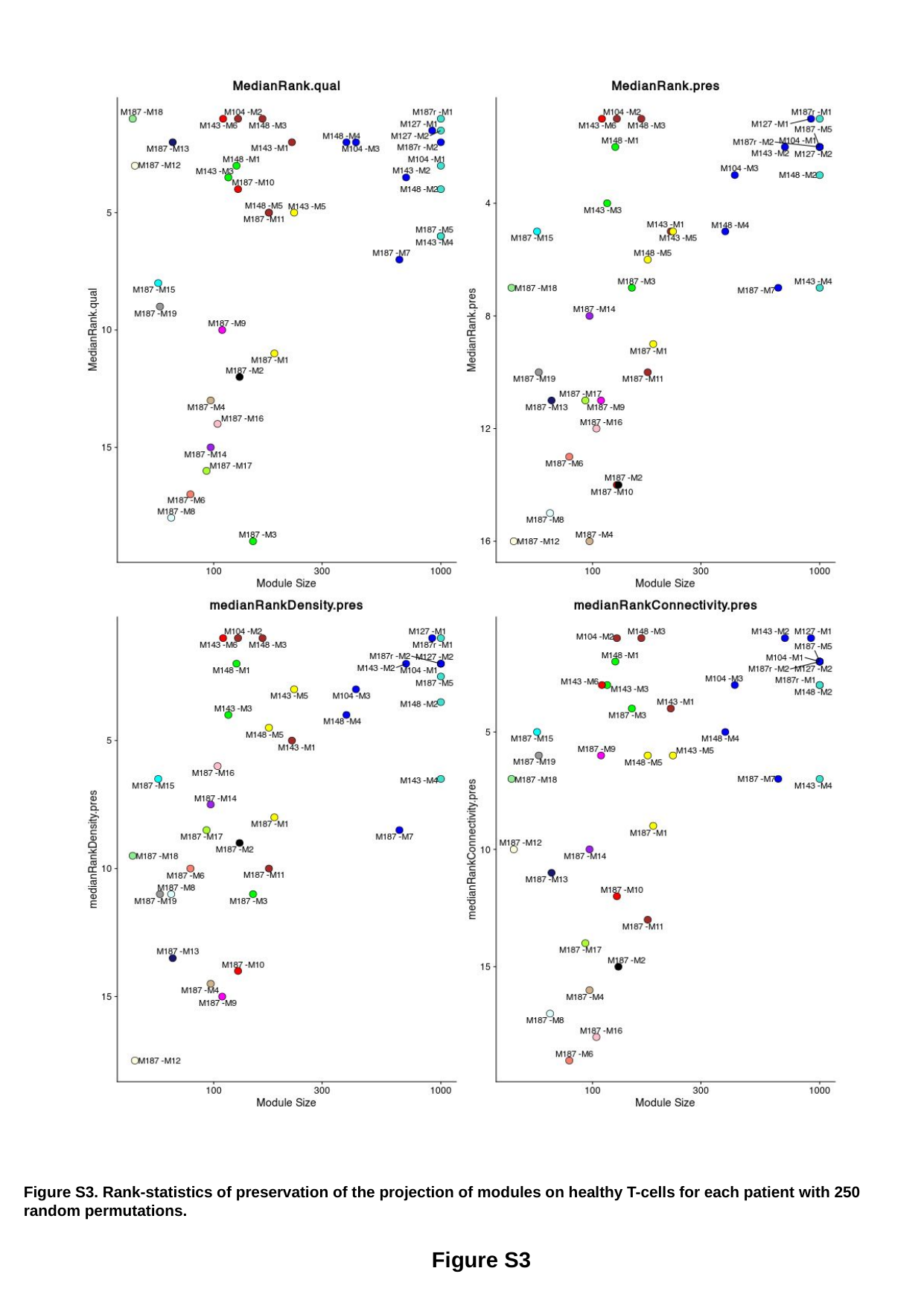

Figure S3. Rank-statistics of preservation of the projection of modules on healthy T-cells for each patient with 250 random permutations.
Figure S3

## Slide 4
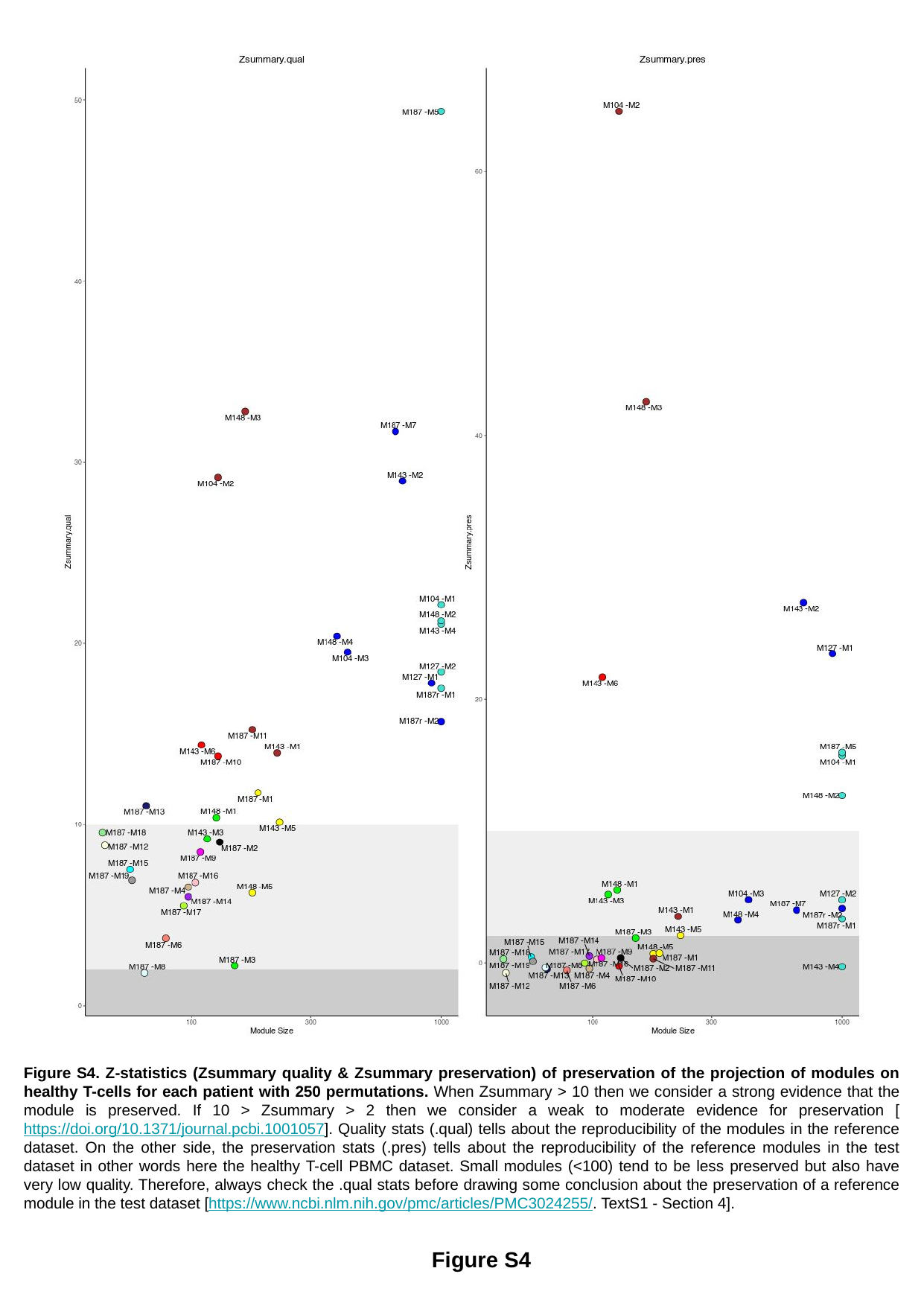

Figure S4. Z-statistics (Zsummary quality & Zsummary preservation) of preservation of the projection of modules on healthy T-cells for each patient with 250 permutations. When Zsummary > 10 then we consider a strong evidence that the module is preserved. If 10 > Zsummary > 2 then we consider a weak to moderate evidence for preservation [https://doi.org/10.1371/journal.pcbi.1001057]. Quality stats (.qual) tells about the reproducibility of the modules in the reference dataset. On the other side, the preservation stats (.pres) tells about the reproducibility of the reference modules in the test dataset in other words here the healthy T-cell PBMC dataset. Small modules (<100) tend to be less preserved but also have very low quality. Therefore, always check the .qual stats before drawing some conclusion about the preservation of a reference module in the test dataset [https://www.ncbi.nlm.nih.gov/pmc/articles/PMC3024255/. TextS1 - Section 4].
Figure S4

## Slide 5
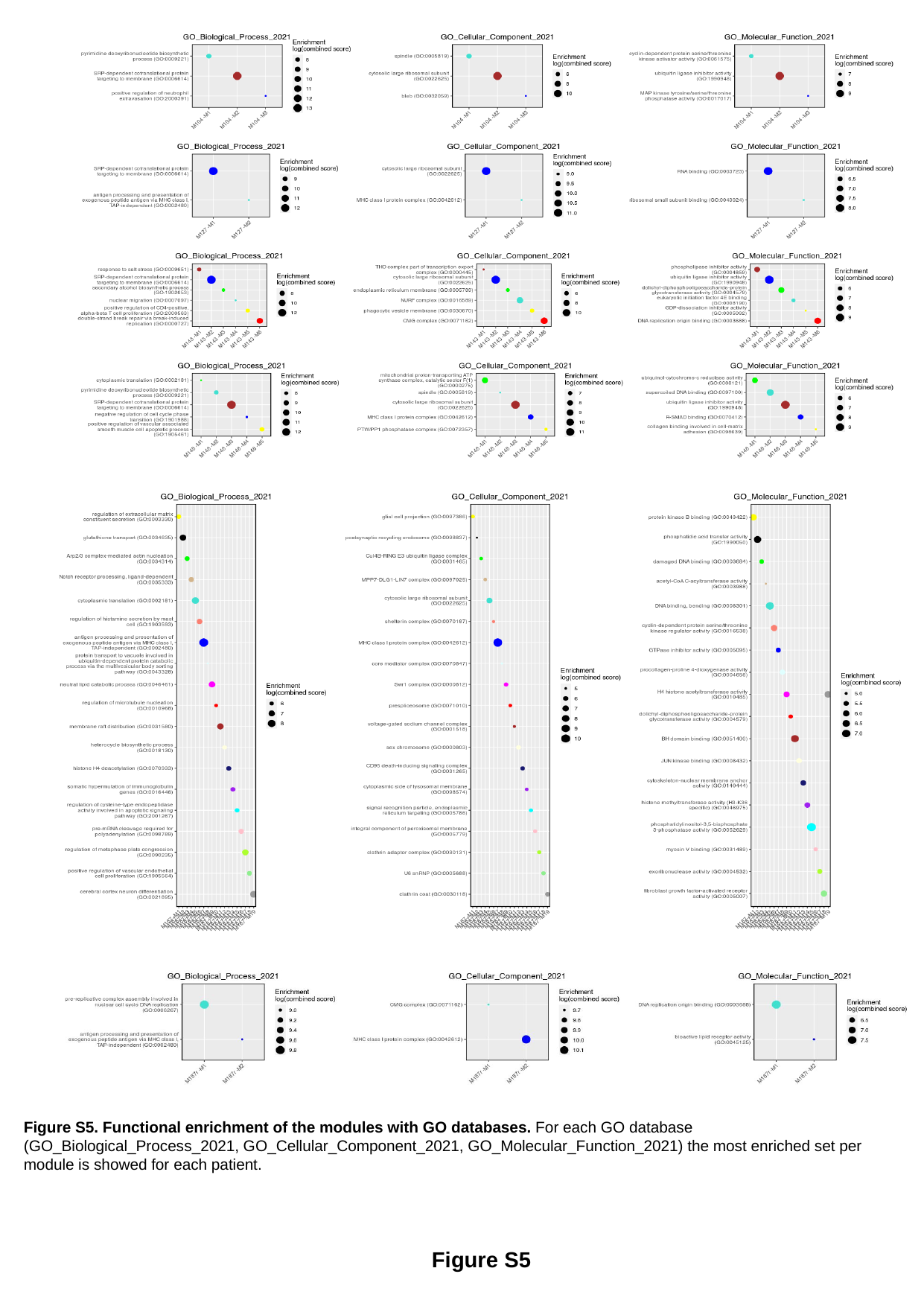

Figure S5. Functional enrichment of the modules with GO databases. For each GO database (GO_Biological_Process_2021, GO_Cellular_Component_2021, GO_Molecular_Function_2021) the most enriched set per module is showed for each patient.
Figure S5

## Slide 6
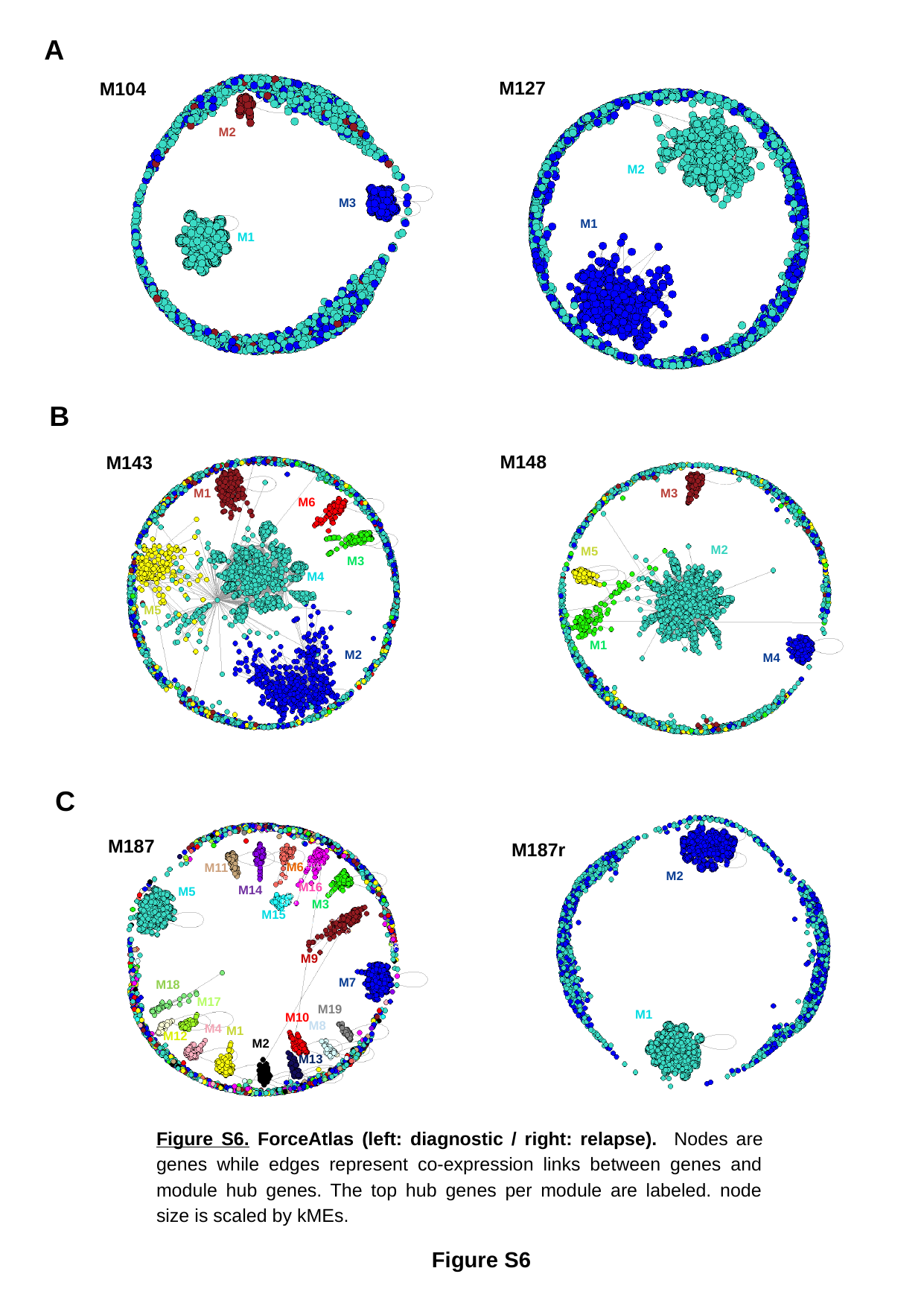

A
M2
M3
M1
M2
M1
M127
M104
B
M1
M6
M3
M4
M5
M2
M3
M2
M5
M1
M4
M148
M143
M2
M1
C
M6
M11
M16
M14
M5
M3
M15
M9
M7
M18
M17
M19
M10
M8
M4
M1
M12
M2
M13
M187
M187r
Figure S6. ForceAtlas (left: diagnostic / right: relapse). Nodes are genes while edges represent co-expression links between genes and module hub genes. The top hub genes per module are labeled. node size is scaled by kMEs.
Figure S6

## Slide 7
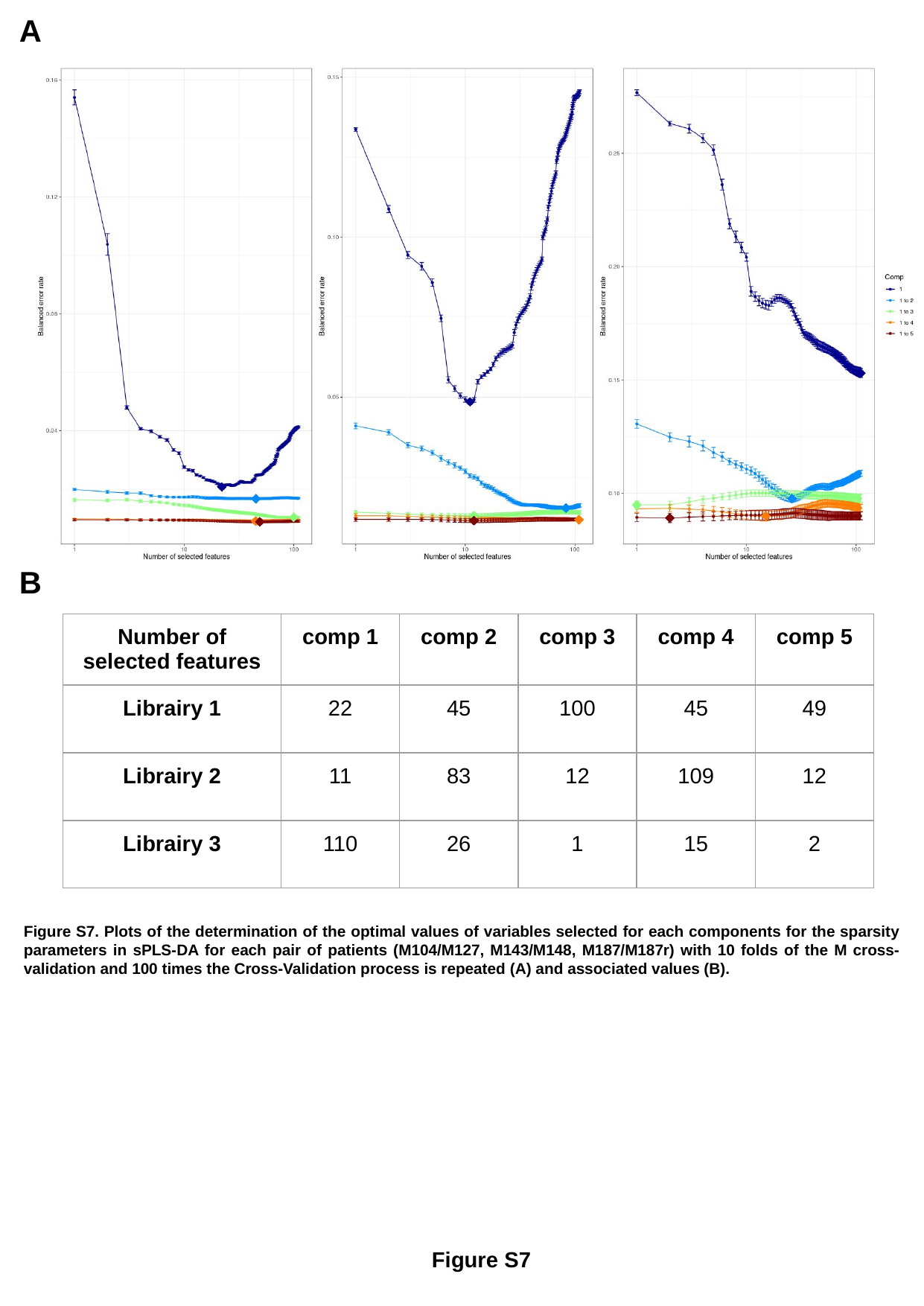

A
B
| Number of selected features | comp 1 | comp 2 | comp 3 | comp 4 | comp 5 |
| --- | --- | --- | --- | --- | --- |
| Librairy 1 | 22 | 45 | 100 | 45 | 49 |
| Librairy 2 | 11 | 83 | 12 | 109 | 12 |
| Librairy 3 | 110 | 26 | 1 | 15 | 2 |
Figure S7. Plots of the determination of the optimal values of variables selected for each components for the sparsity parameters in sPLS-DA for each pair of patients (M104/M127, M143/M148, M187/M187r) with 10 folds of the M cross-validation and 100 times the Cross-Validation process is repeated (A) and associated values (B).
Figure S7

## Slide 8
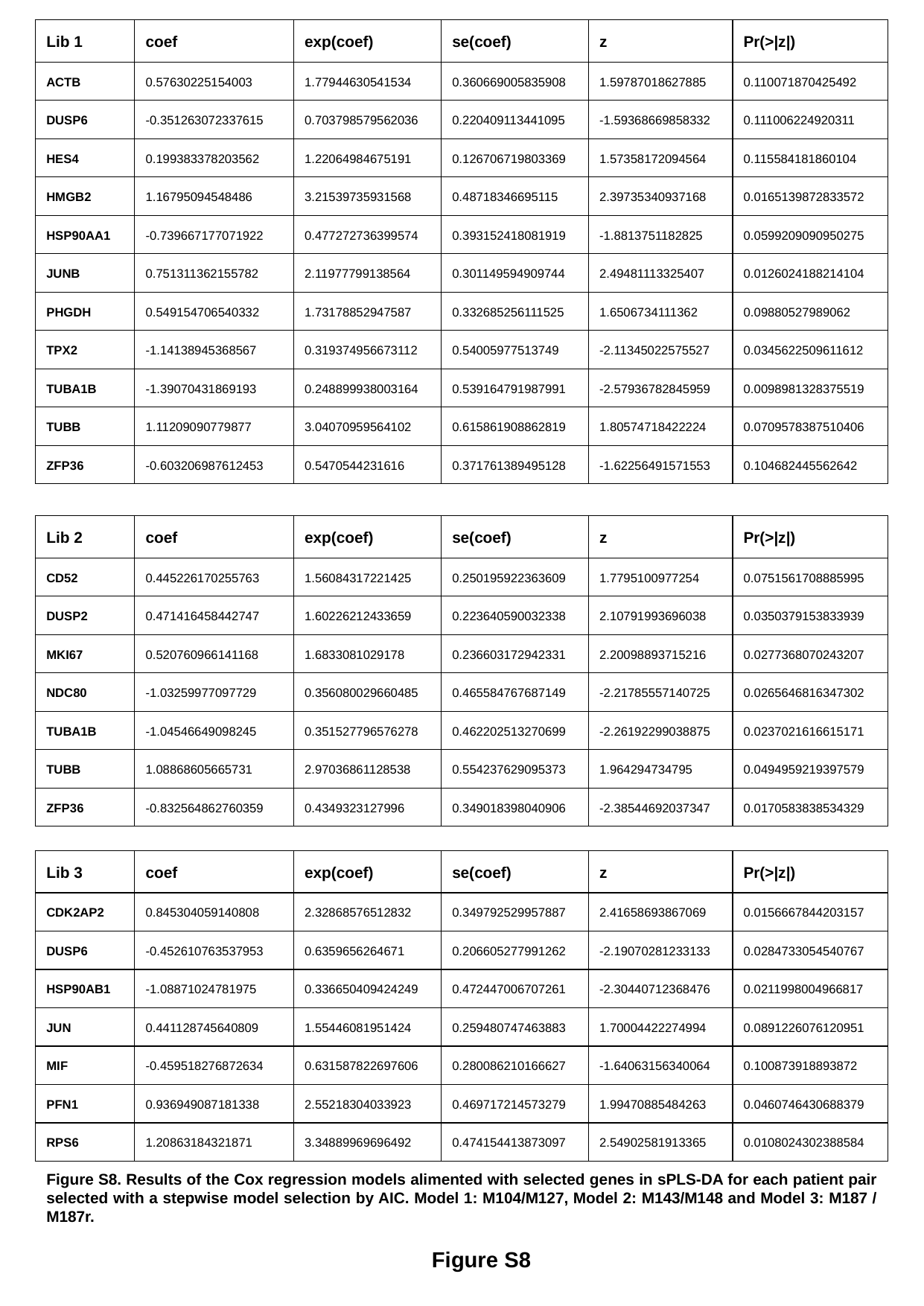

| Lib 1 | coef | exp(coef) | se(coef) | z | Pr(>|z|) |
| --- | --- | --- | --- | --- | --- |
| ACTB | 0.57630225154003 | 1.77944630541534 | 0.360669005835908 | 1.59787018627885 | 0.110071870425492 |
| DUSP6 | -0.351263072337615 | 0.703798579562036 | 0.220409113441095 | -1.59368669858332 | 0.111006224920311 |
| HES4 | 0.199383378203562 | 1.22064984675191 | 0.126706719803369 | 1.57358172094564 | 0.115584181860104 |
| HMGB2 | 1.16795094548486 | 3.21539735931568 | 0.48718346695115 | 2.39735340937168 | 0.0165139872833572 |
| HSP90AA1 | -0.739667177071922 | 0.477272736399574 | 0.393152418081919 | -1.8813751182825 | 0.0599209090950275 |
| JUNB | 0.751311362155782 | 2.11977799138564 | 0.301149594909744 | 2.49481113325407 | 0.0126024188214104 |
| PHGDH | 0.549154706540332 | 1.73178852947587 | 0.332685256111525 | 1.6506734111362 | 0.09880527989062 |
| TPX2 | -1.14138945368567 | 0.319374956673112 | 0.54005977513749 | -2.11345022575527 | 0.0345622509611612 |
| TUBA1B | -1.39070431869193 | 0.248899938003164 | 0.539164791987991 | -2.57936782845959 | 0.0098981328375519 |
| TUBB | 1.11209090779877 | 3.04070959564102 | 0.615861908862819 | 1.80574718422224 | 0.0709578387510406 |
| ZFP36 | -0.603206987612453 | 0.5470544231616 | 0.371761389495128 | -1.62256491571553 | 0.104682445562642 |
| Lib 2 | coef | exp(coef) | se(coef) | z | Pr(>|z|) |
| --- | --- | --- | --- | --- | --- |
| CD52 | 0.445226170255763 | 1.56084317221425 | 0.250195922363609 | 1.7795100977254 | 0.0751561708885995 |
| DUSP2 | 0.471416458442747 | 1.60226212433659 | 0.223640590032338 | 2.10791993696038 | 0.0350379153833939 |
| MKI67 | 0.520760966141168 | 1.6833081029178 | 0.236603172942331 | 2.20098893715216 | 0.0277368070243207 |
| NDC80 | -1.03259977097729 | 0.356080029660485 | 0.465584767687149 | -2.21785557140725 | 0.0265646816347302 |
| TUBA1B | -1.04546649098245 | 0.351527796576278 | 0.462202513270699 | -2.26192299038875 | 0.0237021616615171 |
| TUBB | 1.08868605665731 | 2.97036861128538 | 0.554237629095373 | 1.964294734795 | 0.0494959219397579 |
| ZFP36 | -0.832564862760359 | 0.4349323127996 | 0.349018398040906 | -2.38544692037347 | 0.0170583838534329 |
| Lib 3 | coef | exp(coef) | se(coef) | z | Pr(>|z|) |
| --- | --- | --- | --- | --- | --- |
| CDK2AP2 | 0.845304059140808 | 2.32868576512832 | 0.349792529957887 | 2.41658693867069 | 0.0156667844203157 |
| DUSP6 | -0.452610763537953 | 0.6359656264671 | 0.206605277991262 | -2.19070281233133 | 0.0284733054540767 |
| HSP90AB1 | -1.08871024781975 | 0.336650409424249 | 0.472447006707261 | -2.30440712368476 | 0.0211998004966817 |
| JUN | 0.441128745640809 | 1.55446081951424 | 0.259480747463883 | 1.70004422274994 | 0.0891226076120951 |
| MIF | -0.459518276872634 | 0.631587822697606 | 0.280086210166627 | -1.64063156340064 | 0.100873918893872 |
| PFN1 | 0.936949087181338 | 2.55218304033923 | 0.469717214573279 | 1.99470885484263 | 0.0460746430688379 |
| RPS6 | 1.20863184321871 | 3.34889969696492 | 0.474154413873097 | 2.54902581913365 | 0.0108024302388584 |
Figure S8. Results of the Cox regression models alimented with selected genes in sPLS-DA for each patient pair selected with a stepwise model selection by AIC. Model 1: M104/M127, Model 2: M143/M148 and Model 3: M187 / M187r.
Figure S8

## Slide 9
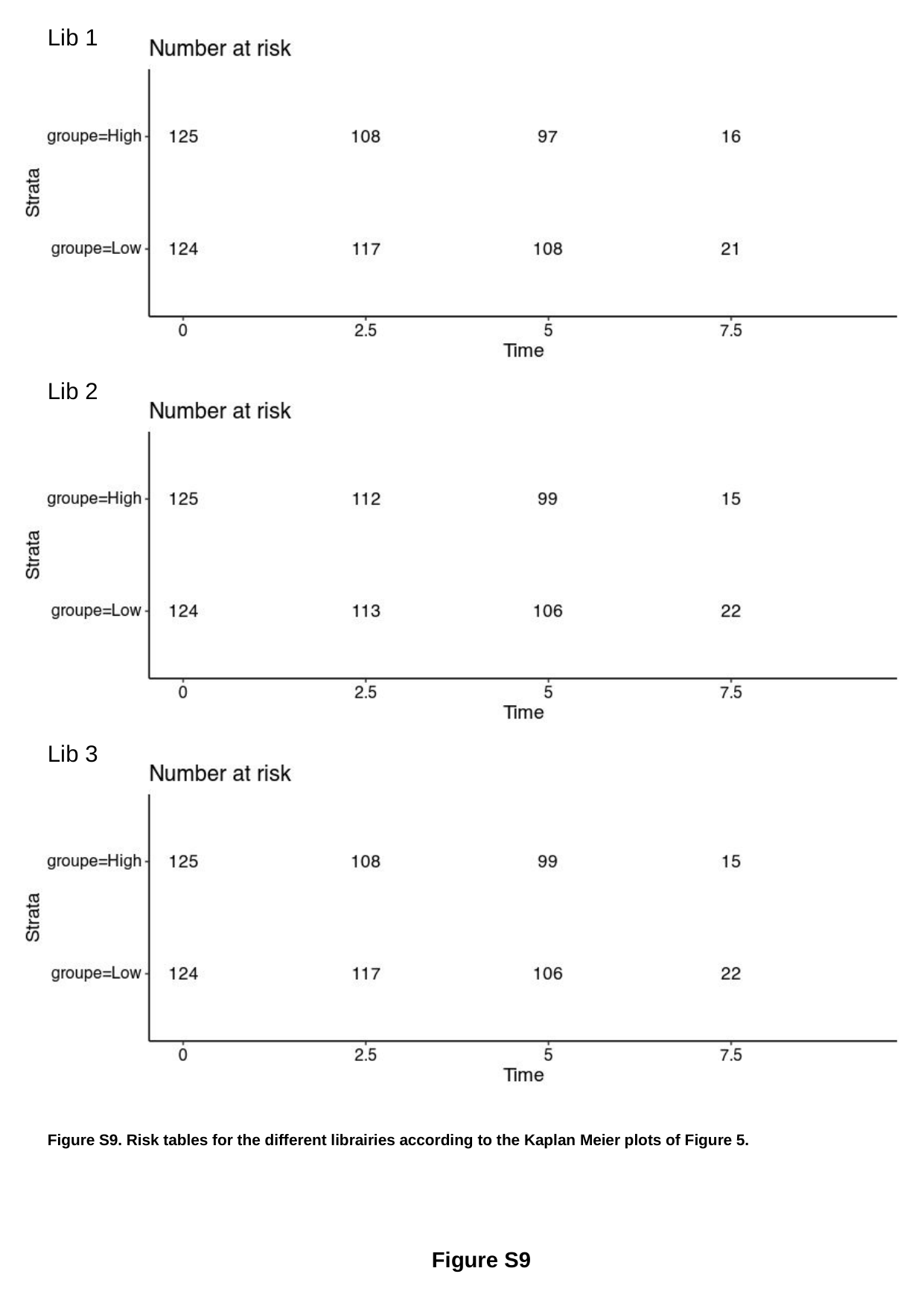

Lib 1
Lib 2
Lib 3
Figure S9. Risk tables for the different librairies according to the Kaplan Meier plots of Figure 5.
Figure S9
